# Nasopharyngeal Carcinoma Ex Pleomorphic Adenoma: Case Report and Comprehensive Literature Review

**DOI:** 10.1155/2021/8892280

**Published:** 2021-02-26

**Authors:** Ellen L. Tokarz, Adian A. Ong, Mark S. Burke

**Affiliations:** State University of New York at Buffalo, Department of Otolaryngology–Head and Neck Surgery, Buffalo, NY, USA

## Abstract

Carcinoma ex pleomorphic adenoma (CXPA) is an epithelial malignancy that transforms from benign pleomorphic adenomas (PA) at a rate of 1.5% after 5 years and 10% after 15 years. The average age of reported nasopharyngeal CXPA is 56.7 years. However, the present case describes a 19-year-old making this case exceptionally rare. Standard treatment is wide local excision with adjuvant treatment. We report the demographics, presentation, treatment, and outcomes of 8 cases of nasopharyngeal CXPA. While surgical excision is the mainstay of treatment, negative margins can be difficult to obtain at the skull base, and we report a recurrence rate of 50% in nasopharyngeal primaries. Due to the aggressive nature of the disease and high rate of recurrence, the majority of patients in our review received adjuvant radiation with some receiving adjuvant chemotherapy in addition.

## 1. Introduction

Carcinoma ex pleomorphic adenoma (CXPA) is a carcinoma arising from a primary or recurrent benign pleomorphic adenoma (PA) and accounts for approximately 12% of all malignant salivary carcinomas. However, its occurrence in the nasopharynx is exceedingly rare [1–3]. CXPA generally occurs in the 5^th^ to 8^th^ decade of life and is more common in females [4]. The later presentation of CXPA has been attributed to the transformation of a long-standing untreated PA, with a rate of transformation ranging from 3%–13.3% [5]. Standard treatment for CXPA is wide local excision with consideration for adjuvant therapy (either radiation and/or chemotherapy). However, the benefit of adjuvant therapy has not been clearly elucidated in the literature. The reported survival ranges from 30% to over 70% depending on stage [6].

Although several studies report the rarity of sinonasal and nasopharyngeal PA and CXPA, to our knowledge, no studies specifically review nasopharyngeal CXPA. The present study aims to report a rare case of nasopharyngeal CXPA in a young adult with review of the literature on previously reported cases of nasopharyngeal CXPA.

## 2. Case Report

A 19-year-old Caucasian female was referred for evaluation of a nasopharyngeal mass. She was initially seen by her primary care physician (PCP) for complaints of bilateral nasal congestion, facial pain, right-sided otalgia, rhinorrhea, and epistaxis for 2.5 months. She was treated by her PCP with antibiotics followed by steroids for several weeks with no improvement. She had persistent symptoms and developed throat pain, dysphagia, snoring, and “throat closing” sensation ultimately leading to otolaryngology referral. Nasal endoscopy by an outside otolaryngologist revealed a large fungating mass emanating from the right nasopharynx extending into the oropharynx. CT scan with IV contrast showed a soft tissue mass 2.5 × 5.1 × 5.9 cm with extension into parapharyngeal, prevertebral, carotid, retropharyngeal, and masticator spaces. The patient was subsequently referred to our institution for further workup. PET scan was performed which showed a bulky FDG avid mass centered in the right nasopharynx with no distant metastasis.

**Figure 1:**
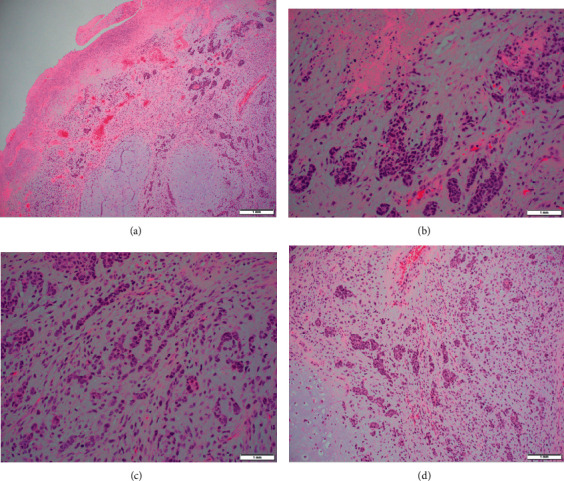
Histological appearance of tumor. (a) HE stain of tumor revealing mucinous and chondromyxoid background with mixed epithelial and myoepithelial differentiation. There is squamous metaplasia of myoepithelial cells and prominent mitotic activity: (b)–(d) higher power HE tumor section.

**Figure 2:**
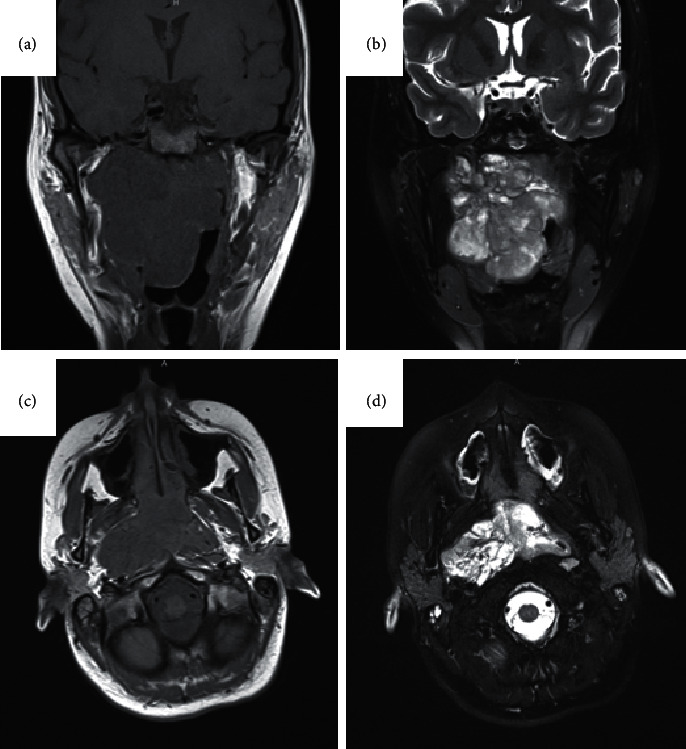
MRI findings of nasopharyngeal mass. (a) T1-weighted MRI in the coronal plane (b) T2-weighted coronal plane (c) T1-weighted axial plane (d) T2-weighted axial plane.

The patient underwent biopsy of the nasopharyngeal mass and was diagnosed with a myoepithelial CXPA (Figure 1). One month after initial presentation to our clinic, she presented to the emergency room with significant oropharyngeal obstruction and severe shortness of breath requiring urgent tracheostomy. Preoperative MRI revealed isointense T1 and hyperintense T2 avidly enhancing mass 7 × 7 × 6.5 cm in the right nasopharynx with extension across midline, inferiorly into the oropharynx, laterally into paraphernal space, and superiorly encroaching the skull base but without evidence of skull base invasion or intracranial extension (Figure 2). One week later, she was taken to the operating room where she underwent excision of the nasopharyngeal mass with right lateral pharyngotomy, right selective neck dissection (levels II, III, and V), right marginal mandibulectomy, and transpalatal approach for nasopharyngeal resection with partial resection of the hard palate and placement of right tympanostomy tube. Her postoperative course was uneventful, and she was decannulated and discharged one week after surgery. Final pathology confirmed myoepithelial CXPA with tumor focally present at the tumor margin, no evidence of lymphovascular or perineural invasion, and no neck metastasis.

Postoperatively, she received 7 weeks of proton beam radiation with weekly cisplatin treatments. After completion of adjuvant therapy, she was without evidence of recurrence until 15 months postoperatively. Unfortunately, at 15 months of postoperation, the patient experienced local recurrence within the retropharyngeal space which was found on surveillance imaging and confirmed with biopsy. She is currently alive with disease at 21 months after initial diagnosis.

## 3. Discussion and Review of Literature

CXPA occurs in major and minor salivary glands. However, its occurrence in the nasopharynx is exceedingly rare. Tumors in the nasopharynx and sinonasal region arise from minor salivary glands in this region and tend to be more aggressive with a higher rate recurrence. However, this is based on very limited available data [2]. Due to the rarity of disease, the exact pathogenesis of transformation is unknown. Further reporting of these tumors will help guide clinicians on treatment options, expected course of disease, and patient counseling.

Only seven previous cases of nasopharyngeal CXPA are reported in the literature, making ours the eighth reported case (Table 1) [1, 7]. The average age at presentation was 50 years with a range from 19–65, and the most common presenting symptom was nasal obstruction. All patients were treated with primary surgical resection, and 87.5% were treated with adjuvant treatment. Of those receiving adjuvant therapy (*n* = 7), two were treated with adjuvant chemoradiation and four were treated with adjuvant radiation alone. The recurrence rate was 50% with an average follow-up time of 2.74 years. At last known follow-up, 2/4 patients with recurrence had died from disease and two were alive with disease.

To our knowledge, our case is the youngest nasopharyngeal CXPA reported in the literature. The average age of patients diagnosed with CXPA, including all head and neck subsites, is 62.1, which is nearly a decade older than our average of 50 years in the nasopharyngeal subsite [6]. CXPA arises from a benign PA, and the overall rate of malignant transformation is 3%–13.3%. However, the incidence increases with time and is 1.5% after 5 years and 10% after 15 years [5, 8]. Our patient likely had a subclinical nasopharyngeal PA as a child or adolescent which transformed into a CXPA and began to rapidly enlarge. To date, there are no reported cases of pediatric patients with confirmed PA who were followed until malignant transformation occurred. Therefore, the rate of malignant transformation of pediatric PA is unknown [5].

Treatment with primary surgical excision is considered the mainstay of treatment [6]. Adjuvant therapy may be used in the form of radiation and/or chemotherapy. However, its effect on overall survival has not yet been determined [6]. In our review of the literature, only one patient did not receive any adjuvant treatment. Of the patients receiving adjuvant therapy, 29% received chemoradiation, while the remaining 71% received adjuvant radiation alone.

We found that positive margins were associated with recurrence in two patients. Yet, in the other two cases, recurrence of margin status was not reported, and we are unable to draw conclusions on the effect of positive margins in nasopharyngeal CXPA recurrence. Margin status in the sinonasal and nasopharyngeal region is difficult to assess due to limited access and frequent piecemeal resection. Furthermore, when tumors abut the skull base or orbit, negative margins may be difficult or impossible to obtain [1]. Other studies reviewing sinonasal and nasopharyngeal CXPA have also been unable to draw reliable conclusions on this correlation [1, 9]. With regard to mortality, Toluie et al. found that disease recurrence in the nasal cavity and nasopharynx was a significant predictor of patients dying from disease. They found that all six patients in their study with recurrence died from their disease. Additionally, all patients in their study with tumor size >4 cm (2/9) died from disease [9]. A recent review by Gupta et al. queried the Surveillance, Epidemiology and End Results (SEER) database to determine predictors of survival for CXPA in all head and neck subsites. Although only 5.2% of tumors reported were outside the major salivary glands, they also found that tumor size >4 cm was a significant predictor of mortality. When considering all head and neck subsites, predictors for mortality were high grade, late stage, distant metastasis, tumor size, extraparenchymal extension, multiple lymph node involvement, and parotid tumors treated with a partial parotidectomy [6].

CXPA diagnosis is made by biopsy with histopathologic diagnosis, but classification can be confusing because tumors are named for their malignant component. The World Health Organization (WHO) recently revised the CXPA tumor classification stating that tumor biology must be determined by the extent of invasion and specific carcinoma subtype [10]. The most common type of CXPA is adenocarcinoma not otherwise specified, followed by salivary duct carcinoma and myoepithelial [11–13]. However, other subtypes do exist and are listed in Table 2. Histological degree of invasion beyond the pleomorphic adenoma further classifies the tumor and is also listed in Table 2 [14–16]. Overall, approximately 90% of CXPA cases are invasive, and the myoepithelial subtype portends the worst prognosis with a high rate of invasive disease [11–13].

This review is limited due to the rarity of nasopharyngeal CXPA. In the future, increased reporting on CXPA disease subsite may help clinicians determine if certain subsites are more aggressive or present at more advanced stages and, in turn, help guide treatment to improve survival. The current review supports evidence that CXPA in the nasopharynx may have a high rate recurrence due to the difficulty in obtaining negative surgical margins. Although CXPA typically presents in the 5^th^-6^th^ decade of life, we report a large, aggressive case occurring in a 19-year-old female. Therefore, CXPA should be considered on the differential diagnosis in patients with a nasopharyngeal mass regardless of age.

## 4. Conclusion

CXPA is an aggressive tumor arising from a benign PA. The mainstay of treatment is surgery with adjuvant chemoradiation. However, there is still a high rate of recurrence and mortality. The disease generally presents later in life and is not typically on the differential diagnosis for young patients with nasopharyngeal masses. However, this report outlines the importance of considering this diagnosis and exploring symptoms of unremitting nasal congestion early, even in young, otherwise, healthy individuals.

## Figures and Tables

**Table 1:** Reported cases of nasopharyngeal carcinoma ex pleomorphic adenoma.

Author (year)	Country	Age (yrs)	Sex	Tumor location	Tumor laterality	Tumor size (cm)	Presenting symptom	Treatment	Tumor pathology	Surgical margins	Recurrence	Follow-up time (months)	Condition at follow-up
Kariya (2006)	Japan	59	F	NP	Right	NS	NO	Neoadjuvant chemo, surgery, adjuvant radiation	Adenoid carcinoma, NOS	N	No	24	DF
Toulie (2012)	US	Mean 51	NS	NP	NS	Mean 3.1	NO, epistaxis	Surgery, adjuvant radiation	Adenoid cystic carcinoma	NS	Yes	NS	DOD
Toulie (2012)	US	Mean 51	NS	NP	NS	Mean 3.1	NO, epistaxis	Surgery, adjuvant radiation	Adenoid cystic carcinoma	NS	Yes	NS	DOD
Li (2019)	China	46	F	NP	Bilateral	2.3 × 1.2	Tinnitus, aural fullness	Surgery, adjuvant radiation	Adenocarcnoma NOS	P	Yes (at 48 mo)	63	AWD
Li (2019)	China	65	M	NP	Bilateral	0.8	Epistaxis	Surgery	Mucoepidermoid carcinoma	N	No	8	DF
Li (2019)	China	49	F	NP	Bilateral	1.2	NO	Surgery, adjuvant radiation	Adenoid cystic carcinoma	N	No	6	DF
Li (2019)	China	58	M	NP	Left	2.5	NO, tinnitus, aural fullness	Surgery, adjuvant radiation	Adenocarcinoma NOS	N	No	8	DF
Present study	US	19	F	NP	Right	7 × 7 × 6.5	NO, epistaxis, aural fullness	Surgery, adjuvant chemoradiation	Myoepithelial carcinoma	P	Yes (at 16 mo)	21	AWD

NS, not specified; NP, nasopharynx; NO, nasal obstruction; NOS, not otherwise specified; N, negative; P, positive; DF, disease free; DOD, died of disease; AWD, alive with disease.

**Table 2:** Classification of CXPA subtypes.

Classification of CXPA subtypes	
Histological cell type [14, 15]	Degree of carcinoma invasion beyond PA [16]

Adenocarcinoma NOS	Noninvasive/intracapsular (confined by tumor capsule)
Salivary duct carcinoma	Minimally invasive (≤1.5 mm beyond the capsule)
Adenosquamous carcinoma	Invasive (>1.5 mm beyond the capsule)
Undifferentiated carcinoma	
Adenoid cystic carcinoma	
Myoepithelial carcinoma	
Epithelial-myoepithelial carcinoma	
Sarcomatoid carcinoma	
Squamous cell carcinoma	
Basal cell adenocarcinoma	
Mucoepidermoid carcinoma	
Oncocytic carcinoma	

## Data Availability

Previously published case reports were used to support this study which are cited at relevant places within the text as references [1, 7, 9].
